# Effect of Different Hosts on Feeding Patterns and Mortality of Mosquitoes (Diptera: Culicidae) and their Implications on Parasite Transmission

**DOI:** 10.4103/0974-777X.62873

**Published:** 2010

**Authors:** Eliningaya J Kweka, Beda J Mwang'onde, Lucile Lyaruu, Filemoni Tenu, Aneth M Mahande

**Affiliations:** *Tropical Pesticides Research Institute, Division of Livestock and Human Disease Vector Control, P.O.Box 3024, Arusha, Tanzania, Tanga*; 1 *National Institute for Medical Research, Amani Research Centre, P. O. Box 81, Muheza, Tanga*

**Keywords:** Feeding pattern, Host behavior, Mortality, Mosquitoes, Parasites

## Abstract

**Aim:**

The host-response to hematophagus insects is still an important parameter in understanding disease transmission patterns. We investigated the feeding and mortality rates of three mosquito species, namely *Culex quinquefasciatus*, *Aedes aegypti* and *Anopheles arabiensis* against three different hosts.

**Materials and Methods:**

Unfed three to five-day-old female mosquitoes were released in a tunnel box that had a rabbit or guinea pig or mice as a host. The feeding succession patterns of mosquitoes in different hosts were *An. arabiensis*, *Cx. quinquefasciatus* and *A. aegypti*.

**Results:**

Mosquito mortality rate was 54.9% for mosquitoes introduced in mice as a host, 34.3% in the Guinea pig and 10.8% for those that introduced in the rabbit.

**Conclusion:**

The presence of defensive and tolerable hosts in environment emphasizes the relevance of studying epidemiological impact of these behaviors in relation to diseases transmission.

## INTRODUCTION

Mosquitoes are among the most active hematophagus insects that transmit diseases by vector-host contact. The observed host vector blood feeding pattern in mosquito varies with species. The ordinary explanation for the observed feeding pattern results from application of the forage ratio technique,[1] which indicates that host preference is dependent upon the relative number of host animals available in the vicinity used by mosquitoes. The environmental factors which the mosquito encounters are important determinants of active host species.[2] Opportunistic response of mosquitoes to hosts influence blood feeding pattern.[3,4]

The body size of the host affects the feeding succession pattern of mosquito species in nature.[5] Large hosts attract big number of mosquitoes, thus considered to be more preferred hosts due to less defensive behavior.[6,7] Host odor plays a major role in attraction of mosquitoes to specific hosts.[8–10] Odor and physiology of individual host may also account for observed variations in mosquito feeding patterns.[9,11] Host defensive behavior in response to feeding of mosquitoes has been suggested to be an important factor in blood feeding succession and parasite transmission.[12] Host activity was mostly responsible for the low feeding succession rates recorded in previous studies.[12,13] Host grooming interrupts blood feeding hence reduces the number of successful blood meals, survivorship and reproduction of mosquitoes.[14,15] Mosquitoes prefer to feed on less defensive vertebrates than those eliciting strong defensive behavior.[16]

Therefore, this study aims to observe the response of host in blood feeding and mortality of three mosquito species against three vertebrate hosts.

## MATERIALS AND METHODS

### Mosquitoes and vertebrates hosts

Mosquitoes used in these experiments were *Aedes aegypti* (L) Kilimanjaro strain, *Anopheles arabiensis* Patton Mabogini strain and *Culex quinquefasciatus* Say, Mabogini strain. All these mosquito species were colonized in the insectary at Tropical Pesticides Research Institute since 1992. Adult females were fed on guinea pig and rabbit for blood meal source in insectary. Three day old unfed female mosquitoes of each species were used in all the experiments. Three vertebrate hosts that were used are rabbit, white laboratory mice and Guinea pig. These hosts are reared at Tropical Pesticides Research Institute, Division of livestock and human disease vectors control for mosquitoes feeding purposes. Each mosquito species was exposed to each vertebrate host.

### Vertebrate exposure to mosquitoes

The unrestrained vertebrate hosts were exposed to mosquitoes by being placed in a 90 cm ×30 cm×30cm glass cage. These cages had five glass sides, with the top open and covered with netting material. Mosquitoes were released in the cage at 18:00hrs and collected at 7:30hrs the following day. The records of mosquito mortality, unfed and fed (partially or fully fed) were taken. In each experimental replica, 25 mosquitoes were used.

### Data entry and analysis

Data was double entered in Ms-access database for validation before analysis using SPSS version 17.0 for windows (SPSS, Inc., Chicago, IL, U.S.A). General linear model univariate analysis was done to assess the effect of host, days of experiment and mosquito species in feeding succession. Excel sheets were used to draw graphs from statistical results values.

## RESULTS

### Mosquitoes feeding in different hosts

**Figure 1:**
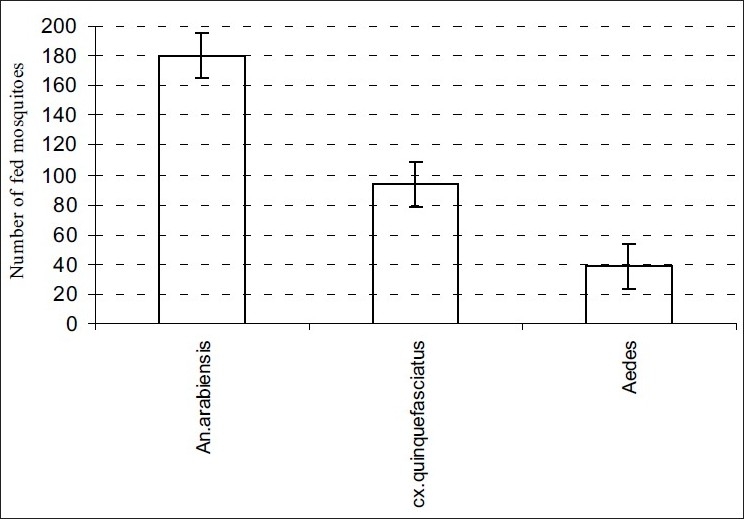
The feeding pattern/response among the three mosquito species to selected vertebrate hosts

**Figure 2:**
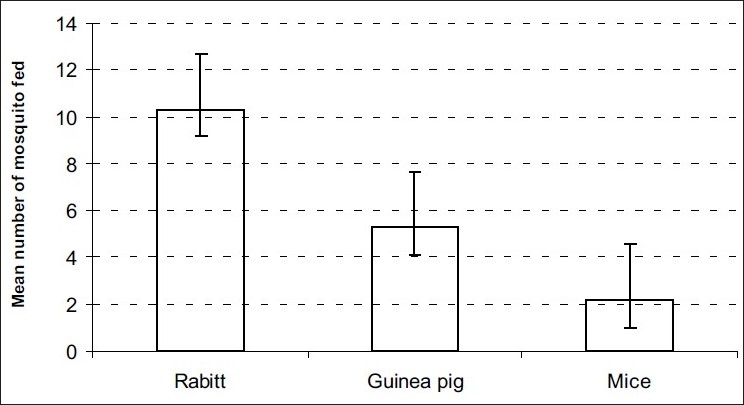
The effect of host body surface area on mosquito-feeding succession

In overall feeding succession, *An. arabiensis* fed most, followed by *Cx. quinquefasciatus* and *Ae. aegypti* was least as shown in Figure 1. Overall, the fed mosquitoes obtained their blood meals from rabbit, guinea pig and least from mice [Figure 2].

### Mosquito mortality

**Figure 3:**
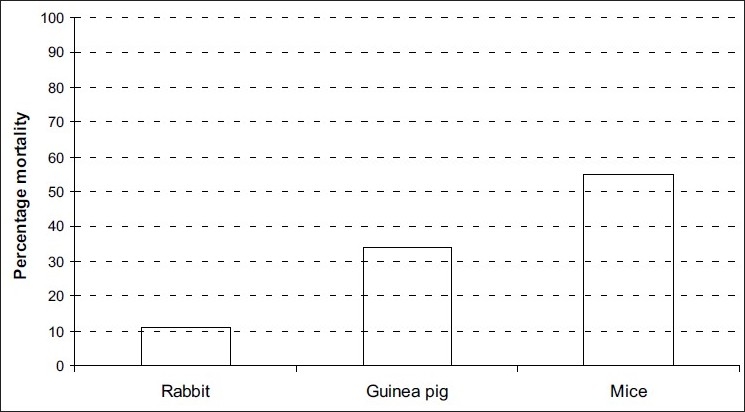
Mosquito mortality as the effect of host surface area size during blood-feeding attempts

Mosquito mortality was observed in all experiments where different hosts where used. Mortality was considered as a result of host defense (animal activity) against mosquitoes probing for blood meal. The overall trend for the mosquito mortality was 54.9% in mice, 34.3% in Guinea pig and 10.8% in rabbits [Figure 3].

## DISCUSSION

The effect of host on the blood feeding pattern and mortality of the mosquitoes found consistent with previous studies.[12,14–16] Results show that feeding pattern remains the same for all mosquitoes for each host species. When different vertebrates are used as baits, large vertebrate host attract more mosquitoes than small ones, this was observed in previous studies.[17,18] When animals are exposed to mosquitoes at the same time (choice experiments), one attracts more mosquitoes than the other due to physiological differences, in agreement with other studies.[8,9,19] It was also observed that mosquitoes fed on rabbit due to its large body surface area than could feed in Guinea pig and mice.[13,18]

Host body surface area might be the factor for mosquito feeding pattern and the strongest selective force leading to the specialized feeding behavior observed. The major changes such as shifting of tolerant vertebrates in natural ecology might have an influence on feeding and parasite transmission rates to man.[13]

Results show that blood meal taking from hosts can be harmful to mosquitoes. The number of mosquitoes recovered in tunnels with mice and guinea pig were extremely lower than those found for rabbit. Mosquito mortality was higher in mosquitoes released in mice and guinea pig tunnels than in the rabbit tunnel because guinea pig and mice are more defensive than rabbit due to their small body surface area. Unfed mosquitoes were as a result of host small body size. In the absence of the intolerant animals (those with small body surface area) the unprotected human population can be at risk of getting bitten by infected mosquitoes. Host body surface area is the most important component in determining the mosquito blood feeding succession and parasites transmission.

## CONCLUSION

Results of this study have demonstrated that body surface area may be diminutive to the influence of the abundance, size and attractiveness of host on mosquito feeding. Zooprophylaxis should be thought in integrated vector management where the malaria vector mosquito species are zoophilic.
